# Impact of alexithymia as a mediator in the relationship between early traumatic experiences and emotion regulation difficulties in young adults

**DOI:** 10.3389/fpsyg.2026.1866103

**Published:** 2026-06-24

**Authors:** Angel Vásquez-Romero, Carlos Miguel Pérez-Lara

**Affiliations:** Department of Psychology, Cesar Vallejo University, Trujillo, Peru

**Keywords:** adverse childhood experiences, affective symptoms, alexithymia, emotion regulation, emotional disorders

## Abstract

**Background:**

Early traumatic experiences have been associated with later difficulties in emotion regulation; however, the underlying mechanisms of this relationship remain insufficiently understood. The objective of the present study was to determine whether alexithymia exerts a mediating role between early traumatic experiences and difficulties in emotion regulation in young adults from Trujillo in 2025.

**Methods:**

The study followed a quantitative approach with a non-experimental, correlational explanatory design of explanatory scope. The sample consisted of 108 young adults, both men and women, aged between 18 and 25 years, from the city of Trujillo, selected through non-probabilistic sampling. The instruments used were the Adverse Childhood Experiences International Questionnaire (ACE-IQ), the Perth Alexithymia Questionnaire (PAQ), and the Cognitive Emotion Regulation Questionnaire (CERQ-27).

**Results:**

Findings indicated that a medium level predominated in early traumatic experiences (62%), alexithymia (56%), and difficulties in emotion regulation (72%). Mediation analysis revealed that alexithymia exerted a significant indirect association (95% CI [0.04, 0.31]) in the relationship between early traumatic experiences and difficulties in emotion regulation.

**Conclusions:**

These results suggest that early trauma is associated with emotion regulation difficulties through alexithymia, highlighting its central role as an underlying psychological mechanism in young adults.

## 1 Introduction

Currently, emotion regulation is regarded as a fundamental skill in the lives of young adults, as it represents a crucial resource for coping with the academic, social, and interpersonal demands characteristic of this developmental stage (Keskiner et al., 2025). However, due to the recent transition from adolescence to young adulthood, a stage marked by significant emotional, social, and identity-related changes; many individuals experience difficulties in emotion regulation, understood as a deficit in managing emotions, encompassing various symptoms associated with intense emotional activation and the inadequate use of strategies to control it (Blancas Guillen et al., 2024). According to a report by the (World Health Organization 2022), in 2019 approximately 970 million people, representing about 13% of the global population, were affected by a mental disorder, with depression and anxiety being the most prevalent. Following the pandemic, the prevalence of these conditions increased by more than 25%. Although there is no specific data on emotion regulation difficulties, these conditions are known to be strongly associated with impairments in emotion regulation, particularly among young adults. Within the framework of SDG 3: Good Health and Well-being, Target 3.4 aims to reduce premature mortality from non-communicable diseases by 33%, prioritizing prevention, access to treatment, and the promotion of mental well-being (Organización de las Naciones Unidas, 2022).

At the international level, difficulties in emotion regulation represent a relevant mental health concern among young adults, as evidenced across different contexts. In Asia, approximately 22.4% of young adults in Indonesia present difficulties in emotion regulation (Danasasmita et al., 2024), while in China, 31.8% report high levels of these difficulties (Hua et al., 2022). In Europe, prevalence rates are also significant, with 38.5% reported in Italy (Guerrini Usubini et al., 2021) and 14.5% in Portugal (Oliveira et al., 2024). In the Latin American context, particularly in Peru, evidence indicates a significant prevalence of emotion regulation difficulties among young adults across different studies conducted at the national level. Reported figures range from 36% to 82.8% (Cardenas Bernardo, 2024; Diaz Mendoza, 2024; More Yarleque and Salvador Cordova, 2024; Seclen Horna, 2024; Lopez Rodriguez and Lopez Villanueva, 2024). Collectively, these findings reflect the widespread nature of this phenomenon within the Peruvian context, suggesting that emotion regulation difficulties are consistently present across diverse sociocultural settings.

International background studies consistently indicate that early traumatic experiences constitute a significant vulnerability factor in the development of psychological distress in young adulthood, with alexithymia and difficulties in emotion regulation serving as central explanatory mechanisms in this relationship. In this regard, studies such as (Karaca Dinç et al. 2021) demonstrate that alexithymia acts as a significant mediator between childhood trauma and psychopathology, explaining a substantial proportion of psychological symptoms, while (Kick et al. 2024), through a meta-analysis, confirm a consistent association between childhood abuse and alexithymia (*r* = 0.26), as well as between alexithymia and psychopathology (*r* = 0.44), highlighting its partial role as a mediating variable. Complementarily, the evidence also underscores the role of emotion regulation as an intermediate mechanism, as (Aydin and Süslü 2023) showed that difficulties in emotion regulation mediate the impact of childhood trauma on psychological resilience, and (Conti et al. 2023) identified that these difficulties account for between 30% and 40% of the variability in post-traumatic stress symptoms. Collectively, these findings converge on the idea that the effect of early traumatic experiences on psychological adjustment in young adults is not direct but is primarily explained by alterations in emotional processing, particularly through alexithymia and difficulties in emotion regulation.

At a national level, the evidence consistently highlights a close interrelationship between alexithymia, emotion regulation difficulties, and contextual psychosocial factors in young adult populations. Specifically, alexithymia has been associated with less adaptive emotion regulation strategies, such as emotional suppression, which in turn is linked to greater psychological distress, including depressive symptomatology (Lewis Paredes, 2021). In parallel, emotion regulation difficulties have been identified as a relevant psychological vulnerability factor that interacts with cognitive-emotional resources such as self-compassion, influencing the intensity of maladaptive responses like fear of negative evaluation (Cardenas Bernardo, 2024). Likewise, familial and parenting environments emerge as key determinants of emotional functioning, with evidence showing that more negative or less supportive family climates are significantly associated with higher levels of alexithymia (Bosio Bobadilla and Cifuentes Cuadros, 2023; Culqui Guevara and Gonzalez Malca, 2024). Overall, these findings converge in suggesting that deficits in emotional awareness and regulation are not isolated phenomena, but rather emerge from the interaction between individual emotional processing capacities and adverse relational contexts, reinforcing the role of alexithymia as a central mechanism in emotional maladjustment among young adults in the national context.

To achieve a clearer understanding of the phenomenon under study, it is necessary to conceptually delimit the variables involved by considering their main theoretical approaches. These conceptual clarifications provide a solid basis for analyzing their relationships and their impact on the target population.

*Early Traumatic Experiences*, are defined as events occurring during childhood and adolescence that significantly disrupt and negatively affect overall developmental processes (Posada Gómez, 2020). (Bellis 2001) further describes them as situations that threaten the physical and/or emotional integrity of the child, including parental neglect, domestic violence, traumatic loss, or highly adverse environments that exceed the emotional coping capacities of both the child and caregivers. These experiences produce immediate consequences that affect behavior and health, leaving a lasting impact on development and increasing the risk of long-term cognitive, emotional, and health-related difficulties (Cohen, 2014). From a theoretical standpoint, the Dimensional Model of Adversity and Psychopathology (DMAP) proposes that childhood adversity is not homogeneous, but rather can be categorized into dimensions such as threat and deprivation. The threat dimension involves exposure to direct harm or danger, which is associated with heightened fear learning, hyperreactivity, and alterations in limbic system functioning. In contrast, the deprivation dimension refers to the absence of adequate cognitive, emotional, or social stimulation, affecting the development of attention and executive control systems.

Within the DMAP framework, the threat dimension refers to experiences in which the child is exposed to imminent and direct risk, leading to heightened sensitivity and activation of fear-related neural circuits. This process contributes to emotional overload, attentional bias toward threatening stimuli, and emotional dysregulation (Miller et al., 2018). On the other hand, the deprivation dimension is associated with a lack of cognitive, emotional, or linguistic stimulation, which negatively affects learning processes and academic performance. It also compromises emotion regulation capacities and increases vulnerability to the development of affective and executive functioning difficulties over time (Miller et al., 2018).

*Alexithymia*, is defined as a stable psychological trait characterized by persistent difficulty in identifying and differentiating feelings from bodily sensations, as well as an impaired ability to describe one's own and others' emotions, accompanied by a tendency toward an externally oriented cognitive style rather than introspective emotional processing (Gonzáles et al., 2025). From this perspective, it is also understood as a reduced capacity to recognize and regulate emotions appropriately in response to environmental demands, which may lead to difficulties in emotional communication and in understanding affective states, both in oneself and in others (Betegón et al., 2022; Gonzáles et al., 2025). In this regard, the interoceptive model of alexithymia proposes that this construct arises from a deficit in the perception and interpretation of interoceptive signals, such as palpitations, tension, and bodily discomfort, which normally enable individuals to recognize and categorize their emotional experiences. This model suggests that such difficulties result from alterations in predictive brain processes that compare internal expectations with bodily signals; when this integration is impaired, individuals experience difficulties in assigning accurate emotional labels to their internal states. Moreover, this framework integrates neurobiological and relational factors, considering the influence of early stressful or traumatic experiences, as well as temperamental predisposition, in the development and maintenance of alexithymia (Lane, 2020; Gaggero et al., 2021; Bael et al., 2024).

Is operationalized through three main dimensions: difficulty identifying feelings (DIF), difficulty describing feelings (DDF), and General-externally oriented thinking (G-EOT). The DIF dimension refers to the inability to recognize and differentiate one's own emotional states, which is associated with a cognitive style marked by rumination, fatalistic thinking, and low cognitive reappraisal capacity. The DDF dimension involves difficulties in verbalizing and expressing emotional states, limiting the use of adaptive coping strategies such as planning and cognitive reappraisal, and instead favoring somatic responses in the absence of emotional articulation. Finally, the G-EOT dimension is characterized by a concrete and externally focused thinking style, with a reduced tendency toward introspection and a preference for practical problem-solving without adequate emotional processing (Betegón et al., 2022).

*Difficulties in emotion regulation*, are conceptualized as persistent limitations in adaptive emotional processes, characterized by an inability to adequately modulate the intensity, duration, and expression of emotions (Thompson, 1994). From this perspective, emotional dysregulation is understood as a deficit in a key component of emotional intelligence, which results in significant difficulties in daily functioning and adaptive coping (Alonso, 2021). These dysfunctions are often rooted in early developmental contexts, particularly in caregiving environments where emotional responses are invalidated, minimized, or censored, limiting the acquisition of effective regulatory strategies during critical stages of emotional learning (Buhler-Wassmann and Hibel, 2021). The Emotion Regulation Process Model further explains that regulatory effectiveness depends on the stage at which intervention occurs within the emotional generation sequence. According to this framework, regulation difficulties emerge early when caregiver–infant interactions lack consistent and sensitive scaffolding, preventing the internalization of adaptive emotional modulation. Over time, these early deficits consolidate through feedback loops characterized by heightened stress reactivity, excessive reliance on maladaptive responses, and the absence of effective coping acquisition, ultimately reinforcing rigid emotional patterns (Paley and Hajal, 2022).

The dimensions of early emotional dysregulation can be organized into clinically and functionally relevant categories. Antecedent-focused adaptive strategies refer to processes such as situation selection, situation modification, attentional deployment, and cognitive reappraisal; their absence reflects poor anticipatory emotional management and limited regulatory flexibility. Response-focused strategies involve mechanisms such as suppression, avoidance, or escape, which, when overused, contribute to greater emotional dysfunction and reduced psychological adjustment. Finally, transversal processes such as emotional awareness refer to the ability to identify, understand, and appropriately select regulatory strategies according to contextual demands; impairment in this domain is associated with greater severity and persistence of emotional dysregulation (Gross, 2002).

From a cognitive perspective, difficulties in emotion regulation may be reflected in the recurrent use of maladaptive cognitive emotion regulation strategies, such as self-blame, rumination, catastrophizing, and blaming others, as well as in a reduced use of adaptive strategies. These cognitive patterns have been associated with poorer emotional adjustment and greater difficulties in managing emotional experiences. Therefore, the assessment of cognitive emotion regulation strategies provides a relevant framework for understanding emotion regulation difficulties.

In line with the approach of the present study, a mediation model is proposed that redefines the understanding of the relationship between early traumatic experiences and difficulties in emotion regulation by positioning alexithymia as the central explanatory mechanism. This framework is conceptualized as the AVR Model of Alexithymia as a Mediator of Early Traumatic Experiences and Emotion Regulation. Within this framework, the impact of childhood trauma is not directly expressed as emotional dysregulation in young adulthood; rather, it is internalized through a progressive disruption in the ability to identify, differentiate, and express one's own affective states. This limitation in emotional processing constitutes a critical turning point in subjective experience, as it constrains the understanding of one's emotions and, consequently, hinders the effective implementation of regulatory strategies. In this sense, alexithymia operates not merely as an intermediary variable, but as a structural axis that reshapes emotional experience, thereby explaining why adverse early experiences ultimately translate into greater difficulties in managing emotions during young adulthood.

Regarding the justification of the study, the results will contribute to expanding knowledge on how early traumatic experiences are related to difficulties in emotion regulation, considering the mediating effect of alexithymia in young adults. This analysis will allow for a better understanding of why some individuals with adverse early experiences develop greater difficulties in identifying, expressing, and managing their emotions, while others are able to adapt more effectively. In this way, the study will contribute to strengthening contemporary theoretical models on the impact of childhood trauma and the emotional factors that mediate or modulate its effects in adulthood. Furthermore, the findings will serve as a basis for mental health professionals to design prevention and intervention programs that are better aligned with the emotional realities of young adults, particularly through strategies aimed at reducing difficulties in emotion regulation by identifying histories of early traumatic experiences and addressing alexithymia, thereby promoting the development of emotional skills that facilitate adaptation and psychological well-being. In addition, this research will provide instruments and procedures supported by evidence of validity and reliability, aimed at rigorously measuring the core variables of early traumatic experiences, alexithymia, and difficulties in emotion regulation in young adults from Trujillo. This methodological contribution will not only enhance the quality of the current study but also support future research seeking to replicate or expand this model in similar contexts, the findings will benefit the young adult population by enabling the design of early detection actions and preventive intervention programs that respond to their real emotional needs, fostering safer and more supportive environments, as well as promoting better emotional well-being, healthier interpersonal relationships, and improved academic and occupational performance.

Based on the aforementioned considerations, the following research question was formulated: Does alexithymia play a mediating role between early traumatic experiences and difficulties in emotion regulation in young adults from the city of Trujillo in 2025?

Accordingly, the general objective of the study was to determine whether alexithymia serves as a mediator between early traumatic experiences and difficulties in emotion regulation in young adults from Trujillo in 2025. In addition, the specific objectives were: (O1) to identify the level of early traumatic experiences in young adults in the city of Trujillo in 2025; (O2) to identify the level of alexithymia in young adults in the city of Trujillo in 2025; (O3) to identify the level of difficulties in emotion regulation in young adults in the city of Trujillo in 2025; (O4) to analyze the association of early traumatic experiences on difficulties in emotion regulation in young adults in the city of Trujillo in 2025; (O5) to analyze the association of early traumatic experiences on alexithymia in young adults in the city of Trujillo in 2025; and (O6) to analyze the association of alexithymia on difficulties in emotion regulation in young adults in the city of Trujillo in 2025.

## 2 Method

### 2.1 Design

The present study is classified as basic research, as it aims to expand theoretical knowledge regarding the variables under study without seeking immediate practical application, in accordance with the guidelines proposed by (OECD and Eurostat 2018), which define basic research as that oriented toward generating new knowledge without commercial or technological purposes. The methodological approach is quantitative, as it uses numerical data and statistical procedures to analyze and interpret relationships between variables, formulate hypotheses, and test them objectively (Hernández Sampieri and Mendoza Torres, 2018a). In this sense, a non-experimental design was adopted, since variables were not deliberately manipulated but observed in their natural context (Ñaupas Paitán et al., 2018). Following the classification proposed by (Ato et al. 2013), the study is framed within a non-experimental correlational explanatory design, aimed at exploring complex relationships between variables and understanding the mechanisms that explain them. Within this framework, a mediation model was employed to analyze both direct and indirect effects, following the approach proposed by (Hayes 2018). Finally, the scope of the research is explanatory, as it seeks to identify causal relationships and understand how the studied variables interact (Hernández Sampieri and Mendoza Torres, 2018a).

### 2.2 Participants

The study population comprised 104,800 young adults (Instituto Nacional de Estadística e Informática (INEI), 2017), defined as individuals sharing specific characteristics from whom the study sample was derived. Inclusion criteria were individuals aged between 18 years and 25 years, of both sexes, who voluntarily agreed to participate. Participants who did not complete the questionnaires or submitted incomplete responses, as well as those who withdrew during the data collection process, were excluded from the study.

The final sample consisted of 108 young adults aged 18 years to 25 years. The sample size was determined using G^*^Power (version 3.1.9.7, Heinrich Heine University Düsseldorf, Düsseldorf, Germany), based on an effect size of *f*^2^ = 0.15, an alpha level of 0.05, statistical power of 0.95, and two predictors, ensuring adequate sensitivity while minimizing Type I and Type II errors. No incentives or compensation were provided for participation. A non-probabilistic purposive sampling technique was employed, selecting participants according to the researcher's judgment and the study's inclusion criteria (Ñaupas Paitán et al., 2018).

### 2.3 Instruments

Data were collected using a structured survey methodology, defined as a systematic procedure that employs standardized questionnaires to obtain information from a sample for the purpose of describing or analyzing attitudes, opinions, or behaviors (Ñaupas Paitán et al., 2018).

Early traumatic experiences were measured using the Adverse Childhood Experiences International Questionnaire (ACE-IQ), a retrospective self-report instrument assessing 13 categories of adversity before the age of 18, with total scores ranging from 0 to 13, where higher scores indicate greater exposure (Santelices et al., 2025). For descriptive purposes, scores were categorized into low, medium, and high levels using percentile-based cut-off points. Previous validation studies report adequate psychometric properties, including good model fit (CFI = 0.98, TLI = 0.97, RMSEA = 0.05, SRMR = 0.05) and acceptable internal consistency (ω = 0.78; α = 0.76).

Alexithymia was assessed using the Perth Alexithymia Questionnaire (PAQ; Preece et al., 2018), a 24-item self-report measure designed to evaluate difficulties in identifying, differentiating, and describing emotional experiences. It comprises five dimensions (N-DIF, P-DIF, N-DDF, P-DDF, and G-EOT) and provides a global score ranging from 24 to 168, where higher scores indicate greater alexithymic traits. For descriptive purposes, scores were categorized into low, medium, and high levels using percentile-based cut-off points. The scale presents strong internal consistency, with subscale reliability ranging from α = 0.87 to 0.91 and overall reliability between α = 0.95 and 0.96.

Cognitive emotion regulation strategies were assessed using the Cognitive Emotion Regulation Questionnaire (CERQ), a 27-item self-report instrument that evaluates cognitive strategies used to regulate emotions, including both adaptive strategies (e.g., positive reappraisal, planning, perspective-taking, and refocusing) and maladaptive strategies (e.g., self-blame, rumination, catastrophizing, and blaming others) (Betegón et al., 2022). The instrument has demonstrated adequate internal consistency, with ω = 0.82 for the total scale, ω = 0.90 for adaptive strategies, and ω = 0.89 for maladaptive strategies. In the present study, difficulties in emotion regulation were conceptualized through the pattern of cognitive emotion regulation strategies assessed by the CERQ-27, considering that a greater predominance of maladaptive strategies is associated with greater difficulties in emotion regulation. For descriptive purposes, scores were categorized into low, medium, and high levels using percentile-based cut-off points.

In the present study, internal consistency was additionally examined, yielding satisfactory reliability coefficients for all variables: Cronbach's alpha (α) and McDonald's omega (ω) exceeded acceptable thresholds (>0.70), supporting the reliability of the instruments in the current sample.

### 2.4 Procedures

The study was conducted in sequential phases to ensure methodological rigor. Initially, a comprehensive literature review was performed to define the study variables and support the formulation of objectives and hypotheses. Subsequently, data were collected using standardized self-report instruments (CERQ-27, ACE-IQ, and PAQ) administered to young adults who met the inclusion criteria, selected through non-probabilistic purposive sampling. Participation was voluntary, and participants received clear instructions to complete the questionnaires honestly and under appropriate conditions. Data quality was then verified by excluding incomplete or inconsistent responses, after which the dataset was coded and prepared for analysis, ensuring anonymity and confidentiality. Finally, statistical analyses were performed to examine the relationships among variables and to test the mediating role of alexithymia.

### 2.5 Statistical analysis

Data analysis was conducted in two sequential stages. First, descriptive statistics were computed using IBM SPSS Statistics version 30 and Microsoft Excel, including frequency distributions, measures of central tendency (mean), dispersion (standard deviation), and tests of normality. The Kolmogorov–Smirnov test with Lilliefors correction was applied to determine data distribution.

Second, inferential analyses were performed based on the assumption of normality. Pearson's correlation coefficient (*r*) or Spearman's rho (ρ) were applied as appropriate. Mediation analysis was conducted using IBM SPSS Statistics version 30 with the PROCESS macro (Hayes), complemented by Jamovi software with the MedMod module, allowing the estimation of direct, indirect, and total effects among the study variables.

### 2.6 Ethical considerations

For the present study, the guidelines established in the (Universidad César Vallejo 2024), which are mandatory for all members of the academic community, were followed. This document outlines principles and criteria aimed at ensuring respect, integrity, and transparency at every stage of the research process. Within this framework, participants' autonomy was guaranteed by providing clear and comprehensible information regarding the objectives, procedures, benefits, and potential risks of the study, as well as their right to withdraw at any time without any negative consequences. Likewise, the confidentiality of the collected information was safeguarded by preserving data anonymity, unless explicit authorization was granted, and by preventing its use or dissemination without proper consent or approval from the ethics committee. The principle of justice was upheld by ensuring fair and non-discriminatory treatment of all participants, regardless of personal, cultural, social, or economic factors. In addition, respect for the dignity, integrity, and cultural worldview of individuals and social groups was maintained, ensuring that the study was conducted with ethical and cultural sensitivity. These principles guided the research team throughout the process, ensuring the protection of participants' rights and well-being, as well as compliance with national and institutional standards governing scientific integrity.

Furthermore, the study adhered to the principles outlined in the Declaration of Helsinki, ensuring the protection of human participants in biomedical and behavioral research. Ethical conduct was also aligned with the standards established by the American Educational Research Association (AERA) and the American Psychological Association (APA, 2018), particularly regarding informed consent, voluntary participation, confidentiality, and responsible data management throughout the research process.

## 3 Results

### 3.1 Descriptive analysis of the data

**Figure 1:**
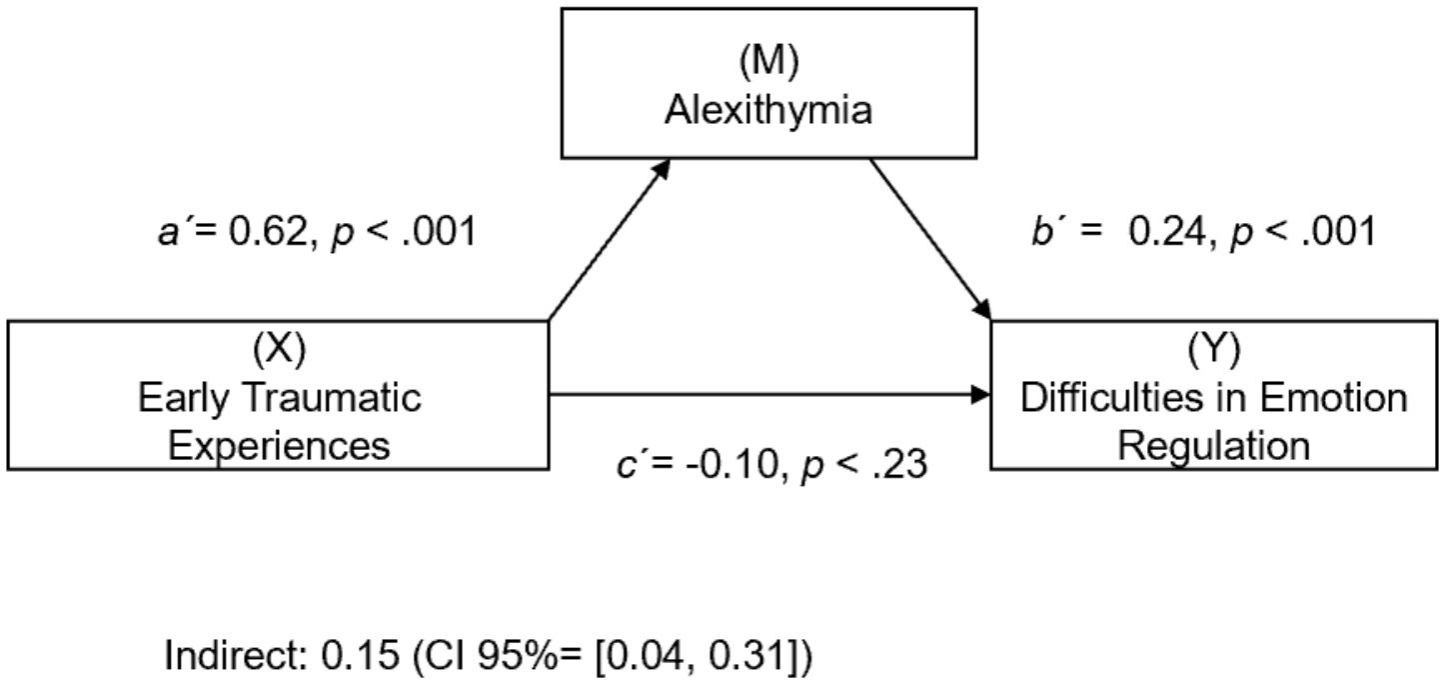
Model diagram.

Table 1 presents the distribution and frequency of the early traumatic experiences variable, where the medium level predominates with 62.0% (*n* = 67), followed by the low level with 38.0% (*n* = 41). In the dimension of relationship with caregivers, the medium level shows the highest proportion with 61.1% (*n* = 66), followed by the low level with 32.4% (*n* = 35). Regarding caregiver neglect, the low level predominates with 50.9% (*n* = 55), followed by the medium level with 46.3% (*n* = 50). In the dysfunctional family environment dimension, the medium level is most frequent with 55.6% (*n* = 60), followed by the low level with 41.7% (*n* = 45). For emotional and physical abuse, the low level predominates with 47.2% (*n* = 51), followed by the medium level with 45.4% (*n* = 49). In the sexual abuse dimension, the medium level is most frequent with 49.1% (*n* = 53), followed by the low level with 47.2% (*n* = 51). In peer violence, the low level predominates with 49.1% (*n* = 53), followed by the medium level with 48.1% (*n* = 52). In community violence, the low level is most frequent with 52.8% (*n* = 57), followed by the medium level with 43.5% (*n* = 47). Regarding exposure to war/collective violence, the low level predominates with 100.0% (*n* = 108). Overall, three out of five participants present a medium level of early traumatic experiences (Figure 1).

**Table 1:** Distribution and frequency of the early traumatic experiences variable.

Level	RC		NC		EFD		AEF		AS		VP		VC		EGV		Early traumatic experiences	
	*n*	*%*	*n*	*%*	*n*	*%*	*n*	*%*	*n*	*%*	*n*	*%*	*n*	*%*	*n*	*%*	*n*	%
High	7	6	3	3	3	3	8	7	4	4	3	3	4	4	0	0	0	0
Medium	66	61	50	46	60	56	49	45	53	49	52	48	47	44	0	0	67	62
Low	35	32	55	51	45	42	51	47	51	47	53	49	57	53	108	100	41	38

*N* = 108. Data extracted from the Adverse Childhood Experiences Scale (ACE-IQ). *RC*, relationship with caregivers; *NC*, caregiver neglect; *EFD*, dysfunctional family environment; *EAF*, Emotional and physical abuse; *SA*, sexual abuse; *PV*, peer violence; *CV*, community violence, *ECV*, exposure to war/collective violence; *ETE*, early traumatic experiences.

Table 2 presents the distribution and frequency of the alexithymia variable, where the medium level predominates with 56.1% (*n* = 60), followed by the low level with 31.8% (*n* = 34). In the dimension of difficulty identifying negative emotions, the medium level shows the highest proportion with 45.5% (*n* = 60), followed by the low level with 29.5% (*n* = 39). Regarding difficulty identifying positive emotions, the medium level predominates with 41.5% (*n* = 56), followed by the low level with 30.4% (*n* = 41). In the dimension of difficulty describing negative emotions, the medium level is most frequent with 51.9% (*n* = 56), followed by the low level with 32.4% (*n* = 35). For difficulty describing positive emotions, the medium level predominates with 37.7% (*n* = 49), followed by the low level with 30.0% (*n* = 39). In the dimension of General externally oriented thinking, the medium level is most frequent with 52.2% (*n* = 60), followed by the low level with 31.3% (*n* = 36). Overall, three out of five participants present a medium level of alexithymia.

**Table 2:** Distribution and frequency of the alexithymia variable.

Level	N-DIF		P-DIF		N-DDF		P-DDF		G-EOT		Alexithymia	
	*n*	*%*	*n*	*%*	*n*	*%*	*n*	*%*	*n*	*%*	*n*	*%*
High	9	6.8	11	8.1	17	15.7	20	15.4	12	10.4	14	13.1
Medium	60	45.5	56	41.5	56	51.9	49	37.7	60	52.2	60	56.1
Low	39	29.5	41	30.4	35	32.4	39	30.0	36	31.3	34	31.8

*N* = 108. Data obtained from the Perth Alexithymia Questionnaire (PAQ). *N-DIF*, difficulty identifying negative emotions; *P-DIF*, difficulty identifying positive emotions; *N-DDF*, difficulty describing negative emotions; *P-DDF*, difficulty describing positive emotions; *G-EOT*, general-externally oriented thinking.

Table 3 presents the distribution and frequency of cognitive emotion regulation difficulties, where the medium level predominates with 72.0% (*n* = 77), followed by the low level with 19.6% (*n* = 21). In the self-blame dimension, the medium level shows the highest proportion with 65.4% (*n* = 70), followed by the low level with 31.8% (*n* = 34). Regarding acceptance, the medium level predominates with 50.0% (*n* = 54), followed by the low level with 25.9% (*n* = 28). In the rumination dimension, the medium level is most frequent with 58.9% (*n* = 63), followed by the low level with 34.6% (*n* = 37). For positive refocusing, the medium level predominates with 58.9% (*n* = 63), followed by the low level with 28.0% (*n* = 30). In the planning dimension, the medium level is most frequent with 51.9% (*n* = 55), followed by the low level with 28.3% (*n* = 30). In positive reappraisal, the medium level predominates with 62.3% (*n* = 66), followed by the low level with 28.3% (*n* = 30). In the putting into perspective dimension, the low level predominates with 55.7% (*n* = 59), followed by the medium level with 43.4% (*n* = 46). In catastrophizing, the low level is most frequent with 53.8% (*n* = 57), followed by the medium level with 44.3% (*n* = 47). Similarly, in blaming others, the low level predominates with 53.8% (*n* = 57), followed by the medium level with 44.3% (*n* = 47). Overall, seven out of ten participants present a medium level of difficulties in emotion regulation.

**Table 3:** Distribution and frequency of cognitive emotion regulation strategies.

Level	SR		AC		RU		PR		PL		PRR		PP		CA		BO		Difficulties in emotional regulation	
	*n*	*%*	*n*	*%*	*n*	*%*	*n*	*%*	*n*	*%*	*n*	*%*	*n*	*%*	*n*	*%*	*n*	*%*	*n*	*%*
High	4	4%	26	24%	8	7%	15	14%	23	22%	12	11%	3	3%	4	4%	4	4%	10	9%
Medium	70	65%	54	50%	63	59%	63	59%	55	52%	66	62%	46	43%	47	44%	47	44%	77	72%
Low	34	32%	28	26%	37	35%	30	28%	30	28%	30	28%	59	56%	57	54%	57	54%	21	20%

*N* = 108. Data obtained from the Cognitive Emotion Regulation Questionnaire (CERQ-27). *SR*, self-blame; *AC*, acceptance; *RU*, rumination; *PR*, positive refocusing; *PL*, planning, *PRR*, positive reappraisal; *PP*, putting into perspective; *CA*, catastrophizing, *BO*,= blaming others.

Table 4 shows that the variables Early Traumatic Experiences, Alexithymia, and Difficulties in Emotion Regulation, as well as their respective dimensions, do not follow a normal distribution (*p* ≤ 0.05). Based on these results, the non-parametric Spearman's rho test was selected for hypothesis testing

**Table 4:** Descriptive data and normality test.

Variables and dimensions	*M*	*SD*	*K-S*	*p*
Early Traumatic Experiences	64.72	19.46	0.10	0.01
Caregiver relationship	5.15	1.66	0.16	0.00
Caregiver neglect	6.31	2.53	0.16	0.00
Dysfunctional family environment	17.14	5.73	0.13	0.00
Emotional and physical abuse	9.00	3.12	0.10	0.01
Sexual abuse	8.25	3.26	0.16	0.00
Peer violence	6.60	2.10	0.11	0.01
Community violence	6.28	2.36	0.12	0.00
Exposure to war/collective violence	6.00	2.55	0.21	0.00
Alexithymia	89.94	30.70	0.14	0.00
Difficulty identifying negative emotions	14.69	5.57	0.12	0.00
Difficulty identifying positive emotions	14.35	5.59	0.13	0.00
Difficulty describing negative emotions	15.81	5.74	0.14	0.00
Difficulty describing positive emotions	14.90	6.31	0.16	0.00
General Externally oriented thinking	30.20	10.82	0.14	0.00
Emotion Regulation Difficulties	81.42	17.86	0.12	0.00
Self-blame	8.65	2.59	0.19	0.00
Acceptance	9.86	2.95	0.11	0.00
Rumination	8.90	2.60	0.11	0.00
Positive refocusing	9.40	2.91	0.10	0.01
Planning	9.74	3.18	0.17	0.00
Positive reappraisal	9.65	3.12	0.09	0.03
Putting into perspective	9.24	2.62	0.11	0.00
Catastrophizing	8.00	2.92	0.12	0.00
Blaming others	7.98	2.92	0.14	0.00

*N* = 108. *M*, Mean; SD, Standard deviation, *K–S*, Kolmogorov–Smirnov test with Lilliefors significance correction, *p*, significance.

### 3.2 Inferential statistical analysis

Table 5 shows that there is a moderate positive correlation between Early Traumatic Experiences (ETE) and alexithymia (rho = 0.43; *p* < 0.001), indicating that higher levels of early traumatic experiences are associated with higher levels of alexithymia among participants. Likewise, ETE presents moderate and significant positive correlations with alexithymia dimensions, including Difficulty Identifying Negative Emotions (rho = 0.36; *p* < 0.001), Difficulty Identifying Positive Emotions (rho = 0.42; *p* < 0.001), Difficulty Describing Positive Emotions (rho = 0.41; *p* < 0.001), and General Externally Oriented Thinking (rho = 0.47; *p* < 0.001). However, no significant correlation was found with the dimension Difficulty Describing Negative Emotions (rho = 0.16; *p* > 0.05). Regarding cognitive emotion regulation difficulties (DER), ETE shows a low but significant positive correlation (rho = 0.27; *p* < 0.01), suggesting that higher levels of early traumatic experiences are associated with greater cognitive emotion regulation difficulties. Regarding the CERQ dimensions, ETE showed low but significant positive correlations with Rumination (rho = 0.24; *p* < 0.05), Putting into Perspective (rho = 0.29; *p* < 0.01), Catastrophizing (rho = 0.51; *p* < 0.01), and Blaming Others (rho = 0.53; *p* < 0.001). However, no significant correlations were found with Acceptance, Positive Refocusing, Planning, and Positive Reappraisal (*p* > 0.05).

**Table 5:** Spearman's rho correlation test.

Variables	1	1.1	1.2	1.3	1.4	1.5	1.6	1.7	1.8	2	2.1	2.2	2.3	2.4	2.5	3	3.1	3.2	3.3	3	3.5	3.6	3.7	3.8	3.9
1. ETE	–																								
1.1 RC	0.09	–																							
1.2 NC	0.89^**^	0.01	–																						
1.3 EFD	0.95^**^	0.08	0.85^**^	–																					
1.4 AEF	0.87^**^	0.05	0.71^**^	0.85^**^	–																				
1.5 AS	0.90^**^	0.00	0.75^**^	0.85^**^	0.76^**^	–																			
1.6 VP	0.86^**^	−0.01	0.75^**^	0.77^**^	0.72^**^	0.75^**^	–																		
1.7 VC	0.83^**^	−0.03	0.67^**^	0.72^**^	0.67^**^	0.75^**^	0.70^**^	–																	
1.8 EGV	0.85^**^	−0.09	0.79^**^	0.75^**^	0.66^**^	0.73^**^	0.77^**^	0.78^**^	–																
2. ALEX	0.43^**^	0.16	0.45^**^	0.46^**^	0.39^**^	0.35^**^	0.34^**^	0.25^**^	0.34^**^	–															
2.1 N-DIF	0.36^**^	0.12	0.38^**^	0.39^**^	0.32^**^	0.29^**^	0.26^**^	0.21^*^	0.29^**^	0.88^**^	–														
2.2 P-DIF	0.42^**^	0.11	0.42^**^	0.43^**^	0.34^**^	0.36^**^	0.32^**^	0.30^**^	0.34^**^	0.87^**^	0.78^**^	–													
2.3 N-DDF	0.16	0.18	0.19^*^	0.21^*^	0.18	0.08	0.12	0.01	0.06	0.83^**^	0.72^**^	0.62^**^	–												
2.4 P-DDF	0.41^**^	0.09	0.44^**^	0.43^**^	0.29^**^	0.34^**^	0.34^**^	0.23^*^	0.39^**^	0.84^**^	0.66^**^	0.77^**^	0.65^**^	–											
2.5 G-EOT	0.47^**^	0.22^*^	0.49^**^	0.50^**^	0.43^**^	0.38^**^	0.34^**^	0.27^**^	0.35^**^	0.92^**^	0.79^**^	0.78^**^	0.74^**^	0.74^**^	–										
3. DER	0.27^**^	0.45^**^	0.17	0.25^**^	0.24^*^	0.19^*^	0.23^*^	0.11	0.19^*^	0.43^**^	0.34^**^	0.35^**^	0.40^**^	0.37^**^	0.44^**^	–									
3.1 AR	0.19^*^	0.23^*^	0.16	0.23^*^	0.19^*^	0.14	0.09	0.09	0.14	0.50^**^	0.46^**^	0.47^**^	0.46^**^	0.37^**^	0.50^**^	0.61^**^	–								
3.2 AC	−0.02	0.46^**^	−0.13	0.06	0.04	−0.03	−0.07	−0.14	−0.17	0.10	0.09	0.00	0.16	0.01	0.18	0.67^**^	0.42^**^	–							
3.3 RU	0.24^*^	0.31^**^	0.18	0.23^*^	0.14	0.10	0.24^*^	0.12	0.28^**^	0.43^**^	0.33^**^	0.37^**^	0.36^**^	0.42^**^	0.41^**^	0.72^**^	0.50^**^	0.37^**^	–						
3.4 RP	0.063	0.47^**^	−0.02	0.04	0.05	−0.02	0.07	−0.00	0.00	0.21^*^	0.20^*^	0.11	0.27^**^	0.12	0.23^*^	0.79^**^	0.39^**^	0.64^**^	0.54^**^	–					
3.5 PL	0.039	0.45^**^	−0.06	0.03	0.08	0.03	−0.03	−0.03	−0.16	0.19^*^	0.07	0.13	0.28^**^	0.12	0.20^*^	0.68^**^	0.47^**^	0.68^**^	0.35^**^	0.59^**^	–				
3.6 RVP	0.00	0.49^**^	−0.10	0.00	0.05	−0.00	−0.03	−0.06	−0.11	0.12	0.07	0.05	0.14	0.08	0.14	0.69^**^	0.22^*^	0.70^**^	0.32^**^	0.66^**^	0.63^**^	–			
3.7 PP	0.29^**^	0.42^**^	0.16	0.25^**^	0.29^**^	0.24^*^	0.28^**^	0.16	0.16	0.28^**^	0.19^*^	0.24^*^	0.21^*^	0.18	0.35^**^	0.76^**^	0.26^**^	0.57^**^	0.47^**^	0.58^**^	0.49^**^	0.73^**^	–		
3.8 CT	0.51^**^	−0.02	0.52^**^	0.47^**^	0.38^**^	0.41^**^	0.52^**^	0.40^**^	0.57^**^	0.54^**^	0.51^**^	0.55^**^	0.38^**^	0.50^**^	0.51^**^	0.54^**^	0.50^**^	0.00	0.53^**^	0.30^**^	0.11	−0.00	0.34^**^	–	
3.9 CO	0.53^**^	0.00	0.51^**^	0.51^**^	0.40^**^	0.41^**^	0.52^**^	0.39^**^	0.61^**^	0.55^**^	0.50^**^	0.47^**^	0.37^**^	0.56^**^	0.54^**^	0.52^**^	0.37^**^	0.07	0.48^**^	0.22^*^	0.02	0.09	0.34^**^	0.71^**^	–

*N* = 108 ^*^p < 0.05, ^**^p < 0.01, *ETE*, early traumatic experiences, *RC*, relationship with caregivers, *NC*, caregiver neglect, *EFD*,= dysfunctional family environment, *EAF*, emotional and physical abuse; *SA*, sexual abuse, *PV*, peer violence, *CV*, community violence; *ECV*, exposure to war/collective violence; *N-DIF*, difficulty identifying negative emotions, *P-DIF*, difficulty identifying positive emotions; *N-DDF*, difficulty describing negative emotions; *P-DDF*, difficulty describing positive emotions; *G-EOT* = general-externally oriented thinking; *SR*, self-blame, *AC*, acceptance, *RU*, rumination, *PR*, positive refocusing, *PL*, planning, *PRR*, positive reappraisal, *PP*, putting into perspective, *CA*, catastrophizing, *BO*, blaming others.

Table 6 shows that early traumatic experiences do not have a statistically significant association on difficulties in emotion regulation (*p* > 0.05). In contrast, alexithymia has a statistically significant association on difficulties in emotion regulation (*p* < 0.05).

**Table 6:** Mediation analysis table for difficulties in emotion regulation (*Y*).

*Model*	*Coeff*	*se*	*t*	*p*	CI 95%	
					LL	UL
Constant	52.46	5.93	8.83	0.00	40.69	64.24
Early traumatic experiences (X)	0.10	0.08	1.18	0.23	−0.06	0.27
Alexithymia (M)	0.24	0.05	4.58	0.00	0.14	0.35

*N* = 108. *Coeff* , coefficient; se, standard error; *t* = student's *t*; *p* = significance, *LL*, lower limit; *UL*, upper limit.

Table 7 shows that early traumatic experiences have a statistically significant association on alexithymia (*p* < 0.05).

**Table 7:** Mediation effect table when the outcome variable is alexithymia (*M*).

Model	Coeff	*se*	*t*	*p*	CI 95%	
					LL	UL
Constant	49.3441	9.4958	5.1964	0.00	30.51	68.17
Early Traumatic Experiences (X)	0.62	0.14	4.46	0.00	0.34	0.90

*N* = 108. *Coeff* , Coefficient; *se*, Standard error, *t* = student's t, *p* = significance, *LL*, lower limit, *UL*, upper limit.

A mediation analysis was conducted using Hayes' PROCESS macro (Model 4) with a bootstrap procedure of 5,000 samples to evaluate whether alexithymia mediates the relationship between early traumatic experiences and difficulties in emotion regulation. Early traumatic experiences did not show a significant direct effect on difficulties in emotion regulation (β = 0.10, SE = 0.08, *p* = 0.23; 95% CI [−0.06, 0.27]). In contrast, alexithymia significantly predicted difficulties in emotion regulation (β = 0.24, SE = 0.05, *p* < 0.001; 95% CI [0.14, 0.35]). Likewise, early traumatic experiences had a significant effect on alexithymia (β = 0.62, SE = 0.14, p < 0.001; 95% CI [0.34, 0.90]). The bootstrap analysis revealed a significant indirect effect (β = 0.15, SE = 0.06; 95% CI [0.04, 0.31]), as the confidence interval did not include zero. Overall, these findings indicate the presence of a significant indirect association, suggesting that the effect of early traumatic experiences on difficulties in emotion regulation is indirectly associated through alexithymia.

## 4 Discussion

In accordance with the general objective of the present study, which was to determine whether alexithymia plays a mediating role between early traumatic experiences and difficulties in emotion regulation in young adults, the results suggest a statistically significant indirect association. Specifically, early traumatic experiences were indirectly associated with difficulties in emotion regulation (β = 0.15, SE = 0.06; 95% CI [0.04, 0.31]). This suggests that early traumatic experiences may be indirectly associated with emotion regulation difficulties through higher levels of alexithymia. In other words, early trauma was not directly associated with cognitive emotion regulation difficulties; rather, it may be related to difficulties in identifying and describing emotions, which are subsequently associated with greater regulatory difficulties (Table 8). These findings are consistent with those reported by (Karaca Dinç et al. 2021), who identified alexithymia as a significant mediator between childhood trauma and psychopathology in young adults (*b* = 0.40, *p* < 0.05). Similarly, the meta-analysis conducted by (Kick et al. 2024) demonstrated that alexithymia partially mediates the relationship between childhood abuse and psychological symptomatology, consolidating its role as a relevant explanatory mechanism. From the interoceptive model of alexithymia proposed by (Lane 2020), early trauma may be associated with disruptions in interoceptive processing, hindering the identification of internal emotional states. This alteration may interfere with adequate emotional categorization, which in turn may affect the implementation of effective regulatory strategies described in Gross's (2002) emotion regulation model. Overall, the present study confirms the findings of (Karaca Dinç et al. 2021) and (Kick et al. 2024), demonstrating that alexithymia functions as a key psychological mechanism associated with the relationship between early traumatic experiences and difficulties in emotion regulation. This pattern may suggest that young adults exposed to early adversity experience difficulties in emotional awareness before presenting broader regulatory difficulties, highlighting the possible role of emotional processing deficits during this developmental stage. Additionally, these findings suggest that adverse childhood experiences may be associated with emotion regulation difficulties in young adults through impairments in emotional awareness and understanding.

**Table 8:** Direct and indirect associations of early traumatic experiences (*X*) with difficulties in emotion regulation (*Y*).

Model	Coeff	*se*	CI 95%	
			LL	UL
Direct Effect (X → Y)	0.10	0.08	−0.06	0.27
Indirect Effect (X → M → Y)	0.15	0.06	0.04	0.31

*N* = 108. Coeff, coefficient; se, standard error; LL, lower limit; UL, upper limit.

On the other hand, the first specific objective was to identify the level of early traumatic experiences among young adults in the city of Trujillo, 2025. According to the results obtained, a medium level of early traumatic experiences predominated with 62.0% (*n* = 67), followed by a low level with 38.0% (*n* = 41) (Table 1). This indicates that approximately three out of five participants present a moderate level of early traumatic experiences, evidencing that a significant proportion of young adults have been exposed during childhood to adverse situations such as neglect, violence, or dysfunctional family environments. These experiences may influence the individual's emotional development and their way of coping with adverse situations in later stages of life. In this regard, (Aguilar Vasquez and Gonzalez Celis 2024) found that early traumatic experiences are associated with various emotional and psychological difficulties in later developmental stages. Likewise, (Di Paola and Nocentini 2023) reported that young adults who experienced higher levels of childhood trauma show greater difficulties in recognizing, interpreting, and regulating their emotions. According to the Dimensional Model of Adversity and Psychopathology, (Miller et al. 2018) argue that adverse experiences during childhood, particularly those related to contexts of threat or deprivation, can affect the development of emotional and cognitive systems. The findings of the present study are consistent with those reported by (Aguilar Vasquez and Gonzalez Celis 2024) and (Di Paola and Nocentini 2023), as they demonstrate that a considerable proportion of young adults have experienced early traumatic events that may influence their emotional development and the way they manage their emotions in later stages of life.

Similarly, the second specific objective was to identify the level of alexithymia among young adults in the city of Trujillo, 2025. According to the results obtained, a medium level of alexithymia predominated with 56.1% (*n* = 60), followed by a low level with 31.8% (*n* = 34) (Table 2). This indicates that more than half of the participants present moderate difficulties in identifying, understanding, and expressing their emotions, reflecting partial limitations in emotional processing. These difficulties may affect the way young adults recognize and communicate their affective states in everyday or stressful situations. In this regard, (Lewis Paredes 2021) found that university students with higher levels of alexithymia tend to use less adaptive emotional strategies, such as emotional suppression, which increases psychological distress. According to the interoceptive model of alexithymia, (Lane 2020) states that this construct is characterized by difficulties in the perception and interpretation of internal bodily signals, which limits the ability to identify and understand emotions. The findings of the present study are consistent with those reported by (Lewis Paredes 2021), as they show that young adults present moderate difficulties in identifying and expressing their emotions, which may influence the way they process and regulate their affective experiences.

On the other hand, the third specific objective was to identify the level of difficulties in emotion regulation among young adults in the city of Trujillo, 2025. According to the results obtained, a medium level of difficulties in emotion regulation predominated with 72.0% (*n* = 77), followed by a low level with 19.6% (*n* = 21), indicating that approximately seven out of ten participants present moderate difficulties in managing their emotions (Table 3). This suggests that young adults show limitations in the effective use of cognitive strategies to regulate their emotions in adverse situations, which may hinder the modulation of emotional intensity and expression in academic or interpersonal stress contexts. In this regard, (Cardenas Bernardo 2024) found that university students presented moderate levels of difficulties in emotion regulation, which were associated with psychological variables such as self-compassion and fear of negative evaluation. Likewise, (Oliveira et al. 2024) reported that young adults may experience difficulties in emotion regulation that affect their psychological well-being and daily functioning. According to (Thompson 1994), emotion regulation is defined as the individual's ability to modulate the intensity, duration, and expression of emotions. Similarly, the emotion regulation model proposed by (Gross 2002) suggests that regulatory difficulties arise when individuals fail to employ appropriate cognitive strategies to reinterpret or modify emotional experiences. The findings of the present study are consistent with those reported by (Cardenas Bernardo 2024) and (Oliveira et al. 2024), as they demonstrate that a considerable proportion of young adults present moderate difficulties in emotion regulation, reflecting limitations in effectively managing their emotions in response to the demands of this developmental stage.

The fourth specific objective was to analyze the association of early traumatic experiences on difficulties in emotion regulation among young adults in the city of Trujillo, 2025. According to the results obtained, no significant direct effect of early traumatic experiences on difficulties in emotion regulation was found (β = 0.10, SE = 0.08, *p* = 0.23) (Table 6). This indicates that early traumatic experiences do not directly predict difficulties in emotion regulation when the proposed model is considered. From an explanatory perspective, childhood trauma alone is not sufficient to generate regulatory difficulties in young adulthood, suggesting that other psychological mechanisms may be involved in this relationship. In this regard, (Sehgal and Kumar 2023) reported that early traumatic experiences are associated with greater difficulties in emotion regulation due to increased affective reactivity and the use of maladaptive strategies. Likewise, (Di Paola and Nocentini 2023) found that the severity of emotional neglect in childhood is related to greater emotion regulation problems in young adults. According to the Dimensional Model of Adversity and Psychopathology, (Miller et al. 2018) explain that the effects of childhood trauma may manifest indirectly through alterations in emotional and cognitive systems, rather than through a directly observable relationship. The findings of the present study partially differ from those reported by (Sehgal and Kumar 2023) and (Di Paola and Nocentini 2023), as no direct association between early traumatic experiences and difficulties in emotion regulation was observed, suggesting the presence of mediating variables associated with this relationship. This finding may indicate that the relationship between early trauma and emotion regulation is more complex than a direct association and could depend on intermediary emotional processes such as alexithymia.

Similarly, the fifth specific objective was to analyze the association of early traumatic experiences on alexithymia among young adults in the city of Trujillo, 2025. According to the results obtained, a significant effect of early traumatic experiences on alexithymia was found (β = 0.62, SE = 0.14, *p* < 0.001; 95% CI [0.34, 0.90]) (Table 7). This indicates that higher levels of early traumatic experiences are associated with higher levels of alexithymia in young adults, evidencing that exposure to adverse events during childhood are directly associated with difficulties in identifying, describing, and understanding one's own emotions. In this regard, (Karaca Dinç et al. 2021) found that alexithymia plays a significant mediating role between childhood traumatic experiences and psychopathology in young adults, showing that higher levels of trauma are associated with higher levels of alexithymia. Likewise, the meta-analysis conducted by (Kick et al. 2024) reported that adverse childhood experiences are significantly correlated with alexithymia in young adulthood. According to the interoceptive model of alexithymia, (Lane 2020) argues that early traumatic experiences may disrupt the development of interoceptive awareness, thereby hindering the identification and categorization of internal emotional states. The findings of the present study are consistent with those reported by (Karaca Dinç et al. 2021) and (Kick et al. 2024), as they demonstrate that early traumatic experiences are significantly associated with alexithymia in young adults. These findings may suggest that difficulties in emotional identification constitute an important psychological vulnerability among young adults with histories of childhood adversity.

The sixth specific objective was to analyze the association of alexithymia on difficulties in emotion regulation among young adults in the city of Trujillo, 2025. According to the results obtained, a significant effect of alexithymia on difficulties in emotion regulation was found (β = 0.24, SE = 0.05, *p* < 0.001; 95% CI [0.14, 0.35]) (Table 6). This indicates that higher levels of alexithymia are associated with greater difficulties in emotion regulation, evidencing that limitations in the identification and expression of emotions directly affect the individual's ability to adequately manage emotional responses in adverse situations. In this regard, (Akpinar 2024) found that university students with higher levels of alexithymia present greater difficulties in emotion regulation, showing increased use of maladaptive strategies. Likewise, (Preece et al. 2023) reported that alexithymia is associated with less functional regulatory styles, affecting the management of both negative and positive emotions. According to the emotion regulation model proposed by (Gross 2002), the identification and understanding of emotions constitute a fundamental step for the selection and implementation of effective regulatory strategies. The findings of the present study are consistent with those reported by (Akpinar 2024) and (Preece et al. 2023), demonstrating that difficulties in identifying and understanding emotions are associated with emotion regulation problems in young adults. This may explain why individuals with alexithymic traits tend to rely on less adaptive regulatory strategies when facing emotionally stressful situations.

### 4.1 Limitations

The present study has several limitations that should be considered when interpreting the findings. First, the use of a non-probabilistic, purposive sampling method may limit the representativeness of the sample and restrict the generalizability of the results to the broader population of young adults. Future research is encouraged to employ probabilistic sampling techniques to enhance external validity. Second, although the sample size was adequate for the analyses conducted, it may still be limited in terms of extrapolating the findings to other contexts or populations; therefore, the results should be interpreted with caution. It is recommended that future studies include larger and more heterogeneous samples to increase the scope and robustness of the findings. Third, the instruments used were based on self-report measures, which may be subject to biases such as social desirability and retrospective recall, particularly in the assessment of early traumatic experiences. In this regard, future research could incorporate multimethod approaches, such as clinical interviews or external assessments, in order to complement the data obtained and improve measurement accuracy.

### 4.2 Implications

The findings of the present study contribute to a broader understanding of the psychological mechanisms underlying the relationship between early traumatic experiences and difficulties in emotion regulation, suggesting that alexithymia may play a mediating role in this association. This evidence supports and refines existing theoretical models of emotional processing, while also enhancing the understanding of the long-term impact of early trauma during young adulthood.

From a clinical perspective, these results suggest that psychological interventions should not focus exclusively on early traumatic experiences, but also on strengthening emotional identification, understanding, and expression skills associated with alexithymia through emotional awareness training, cognitive reappraisal strategies, and emotion-focused therapeutic techniques. In this regard, alexithymia may represent a key explanatory mechanism in the link between early trauma and emotion regulation difficulties. Therefore, prevention and intervention programs could be designed in university and community mental health settings to reduce emotional dysregulation by addressing alexithymia and promoting emotional recognition and adaptive coping skills.

Furthermore, these findings have relevant implications for both clinical and educational settings, as they may guide psychological assessment processes toward the early detection of traumatic histories and difficulties in emotional processing among young adults. This would facilitate the implementation of comprehensive interventions, such as psychoeducational workshops, emotional skills training, and early psychological screening programs tailored to the emotional needs of this population.

At the level of public policy, the results highlight the importance of strengthening mental health programs aimed at young populations, incorporating strategies focused on the prevention of childhood trauma, early emotional assessment, and the promotion of emotion regulation skills through school- and university-based mental health programs. Such efforts may contribute to improving psychological well-being and quality of life among young adults, who represent the target population of this study.

## 5 Conclusion

The findings indicate that alexithymia may represent a relevant mechanism associated with the relationship between early traumatic experiences and difficulties in emotion regulation among young adults in the city of Trujillo, showing a mediation pattern consistent with indirect associations in which the impact of early trauma is not directly expressed as emotional dysregulation but rather through its association on the ability to identify, understand, and express affective states. Likewise, a predominance of moderate levels was identified in early traumatic experiences, alexithymia, and difficulties in emotion regulation, reflecting a relevant presence of emotional vulnerabilities within the studied population. In this sense, the results confirm that early adverse experiences are associated with higher levels of alexithymia, which in turn significantly increases difficulties in emotion regulation, whereas the direct relationship between early trauma and emotion regulation is not statistically significant, thereby reinforcing the relevance of the proposed model for understanding the underlying psychological processes.

## Data Availability

The raw data supporting the conclusions of this article will be made available by the authors, without undue reservation.
